# Visceral adiposity index and sex differences in relation to peripheral artery disease in normal-weight adults with hypertension

**DOI:** 10.1186/s13293-022-00432-4

**Published:** 2022-05-12

**Authors:** Yumeng Shi, Chao Yu, Lihua Hu, Minghui Li, Wei Zhou, Tao Wang, Lingjuan Zhu, Huihui Bao, Ping Li, Xiaoshu Cheng

**Affiliations:** 1 Department of Cardiovascular Medicine, The Second Affiliated Hospital of Nanchang University, No. 1 Minde Road, Nanchang of Jiangxi, 330006 China; 2 Center for Prevention and Treatment of Cardiovascular Diseases, The Second Affiliated Hospital, Nanchang of Jiangxi, China; 3 Jiangxi Provincial Cardiovascular Disease Clinical Medical Research Center, Nanchang of Jiangxi, China; 4 Department of Cardiovascular Medicine, Peking University First Hospital, Beijing, China; 5 Department of Cardiovascular Medicine, Inner Mongolia People’s Hospital, Hu He Hao Te Shi, China

**Keywords:** Visceral adiposity index, Body mass index, Normal weight, Hypertension, Peripheral arterial disease

## Abstract

**Background:**

Previous studies on the relationship between the visceral adiposity index (VAI) and peripheral arterial disease (PAD) are limited. Therefore, this study explored the relationship between VAI and PAD in normal-weight patients with hypertension.

**Methods:**

A total of 6615 normal-weight patients with hypertension were included in the current study. The VAI, a simple index calculated using blood lipid and waist circumference (WC), can be used as a simple biomarker of body fat distribution. The outcome was PAD, which was defined as present when each side’s ankle–brachial index (ABI) was ≤ 0.90.

**Results:**

A significant positive association was observed between VAI and PAD prevalence. For per unit increment in LnVAI, the adjusted odds ratios (ORs) of PAD for the total participants and males were 1.55 (95% CI 1.15–2.10) and 2.12 (95% CI 1.46–3.07), respectively. However, the VAI was not associated with PAD in female patients with hypertension (OR 1.28; 95% confidence interval [CI] 0.85–1.95). There was no interaction between sex and VAI (*P* for interaction = 0.128). Accordingly, in total participants, when VAI was assessed in quartiles and compared with quartile 1 (< 0.84), the PAD prevalence was higher than that of quartiles 2 (0.84 to < 1.36: OR 1.49; 95% CI 0.92–2.44), 3 (1.36 to < 2.25: OR 1.95; 95% CI 1.14–3.32), and 4 (≥ 2.25: OR 1.93; 95% CI 1.04–3.57). There were no significant interactions with the other confounders.

**Conclusion:**

This study showed a positive association between VAI and PAD in normal-weight adults with hypertension among men but not among women.

## Highlights

This study is the first to find a significant positive association between the baseline visceral adiposity index (VAI) and peripheral arterial disease (PAD) in normal-weight adults with hypertension. The present study is also the first to indicate a stronger linear positive relationship between VAI and PAD in male participants but not in females.

## Introduction

Peripheral arterial disease (PAD) is a significant global health problem that affects nearly 10% of people globally, among whom nearly 15–20% over 70 years of age are affected [1, 2]. PAD is characterized by narrowing and obstruction of the peripheral arteries, and the main pathogenic factor is arteriosclerosis [3]. When compared with patients without PAD, patients with PAD are more likely to experience myocardial infarction (MI), stroke, and cardiovascular death [4–7]. Moreover, related studies have reported that blood pressure is closely related to the risk of PAD. The risk of PAD increases with increased blood pressure [8, 9].

An increasing number of observational studies have shown a significant positive correlation between obesity, defined by body mass index (BMI), and PAD [10–12]. Obesity is a metabolic disease. However, some normal-weight people (normal BMI) may have substantial metabolic disorders similar to those with obesity. These individuals were metabolically obese with normal weight. At the same time, related studies show that metabolically obese with normal-weight individuals account for 20% of the normal-weight population [13, 14]. When compared with BMI, visceral fat can better reflect metabolic changes [15]. The visceral adiposity index (VAI), a simple index calculated using blood lipid, waist circumference (WC), and BMI, can be used as a simple biomarker of body fat distribution and metabolic disorder and is closely related to visceral fat measured using magnetic resonance imaging (MRI) [16]. However, the correlation between VAI and PAD has only been carried out in patients with diabetes [17], while the influence of VAI on PAD has seldom been studied in normal-weight patients with hypertension.

To fill this knowledge gap, our current study aimed to evaluate the relationship between VAI level and PAD prevalence rate in normal-weight patients with hypertension using the data from the China H-type Hypertension Registry Study and further explore the possible effect modifiers between them.

## Methods

### Study population

The study was approved by the ethics committees of the Institute of Biomedicine, Anhui Medical University, and the Second Affiliated Hospital of Nanchang University. All participants provided written informed consent.

This study used the data from the China H-type Hypertension Registry Study (registration number: ChiCTR1800017274). The study design and methods have been described previously [18, 19]. Briefly, the China H-type Hypertension Registry Study is an ongoing observational real-world study in Wuyuan, China, from March 2018 to August 2018. The inclusion criteria were patients with hypertension over 18 years of age, hypertension defined as systolic blood pressure (SBP) ≥ 140 mmHg or diastolic BP (DBP) ≥ 90 mmHg at screening visits or if the individual was on antihypertensive medication. The exclusion criteria were psychological or nervous system impairment resulting in an inability to provide informed consent, inability to follow-up according to the study protocol or plans to relocate in the near future, and patients who were not suitable for inclusion or for long-term follow-up as assessed by study physicians.

**Fig. 1:**
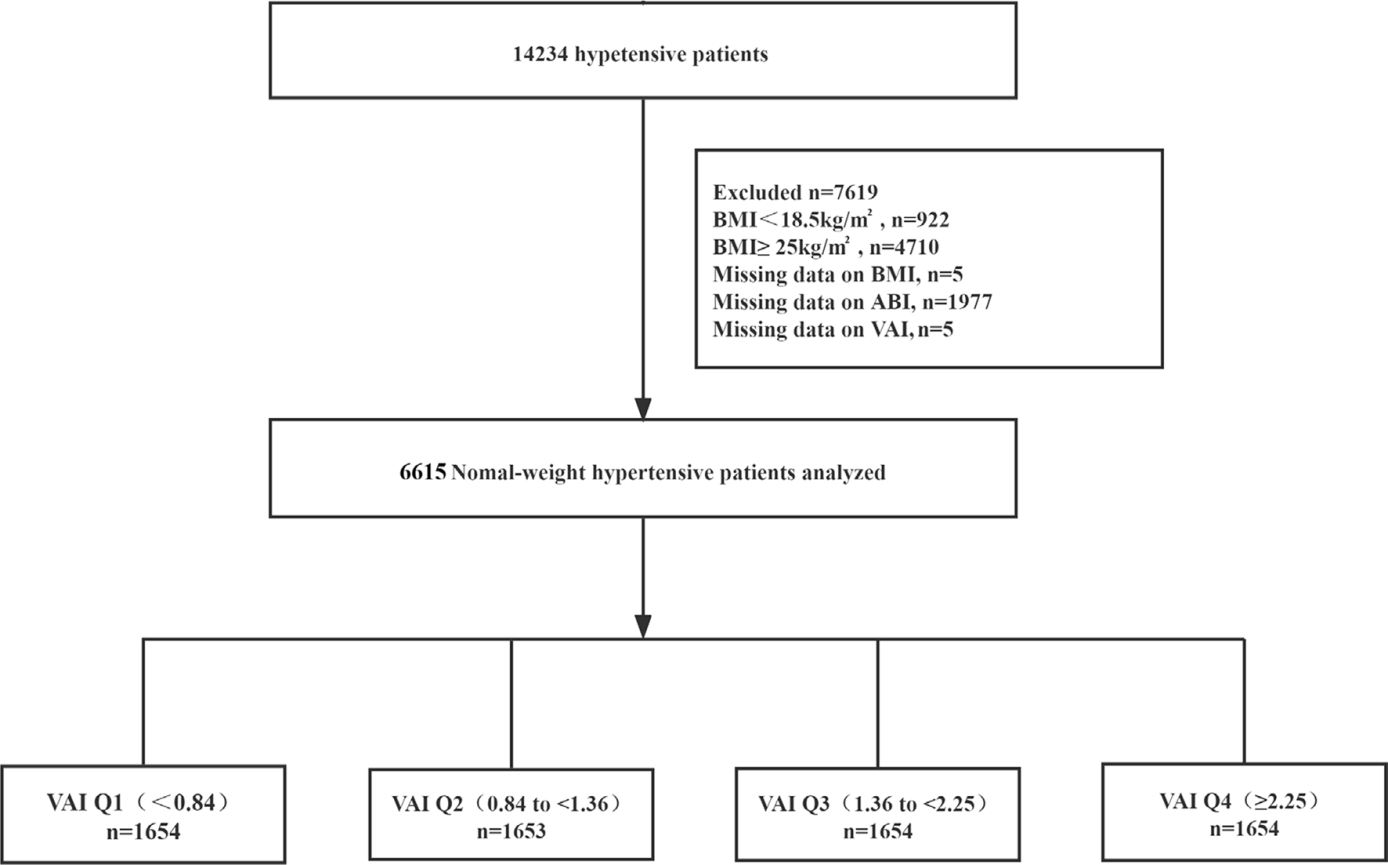
Flow chart of study participants

A total of 14,234 patients with hypertension satisfied the inclusion and exclusion criteria. In our cross-sectional analysis, participants with an abnormal weight according to the World Health Organization (WHO) standards [20] (BMI ˂ 18.5, *n* = 922; BMI ≥ 25, *n* = 4710) and patients with lost BMI (*n* = 5), ankle–brachial index (ABI) (*n* = 1977), and VAI data (*n* = 5) were excluded. Eventually, 6615 normal-weight patients with hypertension were included in the final analysis (Fig. 1).

### Data collection

Trained medical staff performed a health interview using a validated questionnaire to collect the demographic and behavioral characteristics of the study population. Information on demographic and behavioral characteristics included age, sex, education level (less than high school, and at least high school), physical activity, smoking and drinking status, diabetes history, stroke history, and medication information (antihypertensive, lipoprotein-lowering, and glucose-lowering drugs). The current smoking was defined as smoking ≥ 1 cigarette per day for 1 year or more or a cumulative smoking amount ≥ 360 cigarettes per year. Alcohol consumption was defined as drinking an average of two or more times per week over a year. There are two types of drinking: occasional and regular. Occasional drinking was defined as drinking alcohol monthly or less, and regular drinking was defined as drinking alcohol at least twice a month. According to the participants’ evaluation, physical activity was classified as mild, moderate, or vigorous.

Anthropometric data, body height, and WC were measured to the nearest 5 mm by directly touching the participant’s skin using cloth tape. Furthermore, BP was assessed by trained medical staff to limit interobserver variability in the measurements. After the participants had rested for 5 min, seated BP was measured using an electronic sphygmomanometer (Omron; Dalian, China) following the standard method and appropriately sized cuffs. Three measurements on the right arm were performed at 1-min intervals between successive readings, and the mean value was calculated. BMI was defined as body weight/height^2^ (kg/m^2^). In the present study, normal weight was defined as a BMI of 18.5–24.9.

Fasting blood samples were obtained from all patients. All biochemical measurements were conducted at the Biaojia Biotechnology in Shenzhen, Guangdong Province, China, using automatic clinical analyzers (Beckman Coulter, USA). Biochemical data, including fasting plasma glucose (FPG), homocysteine (Hcy), triglyceride (TG), total cholesterol (TC), high-density lipoprotein cholesterol (HDL-C), low-density lipoprotein cholesterol (LDL-C), serum creatinine, aspartate aminotransferase, alanine aminotransferase, and gamma-glutamyl transferase, were obtained from fasting blood samples. According to the Chronic Kidney Disease Epidemiology Collaboration (CKD-EPI) equation, we calculated the estimated glomerular filtration rate (eGFR) [21].

### Definition of the VAI and PAD

The VAI was calculated according to the definition of Amato et al. [22] using sex-specific formulas as follows: females: VAI = [WC/(36.58 + (1.89*BMI))]*(TG/0.81)*(1.52/HDL); males: VAI = [WC/(39.68 + (1.88*BMI))]*(TG/1.03)*(1.31/HDL). In the above formula, the unit of WC is cm, and HDL-C and TGs are mmol/L. After 10 min of rest, an Omron Colin BP-203RPE III device (Omron Health Care, Kyoto, Japan) was used to measure the ABI, with the participant in a supine position, calculated as the highest SBP at the ankles divided by the highest SBP of the right or left upper arms [23]. The data regarding the validity and reproducibility of this automatic device have been published previously [24]. Therefore, the lowest ABI value was used for this analysis. PAD was defined as an ABI ≤ 0.90 in either leg [6].

### Other definition

Diabetes mellitus was defined as self-reported physician diagnosis of diabetes, FBG concentration ≥ 7.0 mmol/L, or use of glucose-lowering drugs. The medical history of stroke was self-reported and was mainly collected using a questionnaire. Each participant was asked whether there was a stroke, when the stroke occurred, symptoms, what kind of treatment was administered, and whether there were relevant medical records, including discharge summary and imaging data. The medical history of coronary heart disease (CHD) was self-reported and was mainly collected using a questionnaire. Each participant was asked whether there was a CHD, when the CHD occurred, symptoms, what kind of treatment was administered, and whether there were relevant medical records, including discharge summary and imaging data.

### Statistical analysis

The baseline characteristics of the study participants were presented as mean ± standard deviation (SD) for continuous variables and percentage (%) for categorical variables by VAI quartiles. Accordingly, differences in population characteristics by VAI quartiles were compared using an one-way analysis of variance or *χ*^2^ tests.

Owing to its left-skewed distribution, the VAI was analyzed after Ln-transformation and quartiles. The VAI was assessed using quartiles and continuous variables. Multivariate logistic regression models were used to evaluate the association between VAI and PAD in normal-weight participants with hypertension. Covariates were included as potential confounders in the final multivariate logistic regression models if the estimates of VAI on PAD changed by more than 10% [25] or were known as traditional risk factors for PAD. Four multivariate regression models were considered: Model 1: age and sex; Model 2: age, sex, BMI, SBP, and DBP; Model 3: diabetes mellitus, stroke, CHD, antihypertensive drugs, lipoprotein-lowering drugs, glucose-lowering drugs, current smoking, current drinking, education, and physical activity, in addition to the variables in Model 2; and Model 4: Hcy, FBG, TC, LDL, eGFR, serum aspartate aminotransferase, alanine aminotransferase, and gamma-glutamyl transferase, in addition to the variables in Model 3. We used a generalized additive model and fitted smoothing curve (penalized spline method) to assess the dose–response association between VAI and PAD prevalence. Stratification analyses according to sex (male vs. female), age (< 60 vs. ≥ 60), physical activity (mild, moderate, or vigorous), current smoking status (no vs. yes), drinking status (no vs. yes), diabetes mellitus (no vs. yes), SBP (< 140, 140–159, or ≥ 160 mmHg), LDL-C (< 2.6 vs. ≥ 2.6 mmol/L), and antihypertensive drugs (no vs. yes) were performed to test whether these factors could modify the association between VAI and PAD, tested by adding a cross-product term between covariates and VAI to the model.

A two-sided *P* value < 0.05 was statistically significant in all analyses. Statistical analyses were performed using the statistical packages R (R Foundation for Statistical Computing, http://www.r-project.org) and Empower (R) (X&Y Solutions, Inc.; www.empowerstats.com).

## Results

### Study participants and baseline characteristics

A total of 6615 normal-weight participants with hypertension with complete ABI and VAI data were included in the final data analysis (Fig. 1). The study population included 3195 (48.30%) males with an average age of 64.84 (SD, 8.80) years. The VAI medians (interquartiles [IQRs]) were 1.36 (0.84–2.25). Of these, 212 (3.20%) participants had PAD, 1043 (15.77%) had diabetes, and 439 (6.64%) had stroke.

The baseline characteristics of the study participants by VAI quartiles are presented in Table 1. Participants with higher VAI had higher values of BMI, pulse rate, FPG, TG, LDL-C, serum alanine aminotransferase, and serum gamma-glutamyl transferase and had a higher prevalence of diabetes mellitus and the use of antihypertensive, lipoprotein-lowering, and glucose-lowering drugs. Furthermore, participants in Q4 (VAI ≥ 2.25) were more likely to be young, female, nonsmoker, and nondrinker, and had lower physical activity levels, Hcy levels, TC, HDL-C, and serum aspartate aminotransferase. However, we found no significant differences in education level, SBP, DBP, CHD, eGFR, and stroke among the VAI quartiles (*P* > 0.05).

**Table 1:** Baseline characteristics of normal-weight hypertensive patients according to VAI

Variable	Ln VAI				*P* value
	Quartile 1	Quartile 2	Quartile 3	Quartile 4	
VAI range	< 0.84	0.84 to < 1.36	1.36 to < 2.25	≥ 2.25	
Participants	1654	1653	1654	1654	
Males, *N*	1302 (78.72%)	839 (50.76%)	626 (37.85%)	428 (25.88%)	< 0.001
Age, year	66.05 ± 8.45	65.67 ± 8.92	64.53 ± 8.82	63.12 ± 8.70	< 0.001
BMI, kg/m^2^	21.47 ± 1.72	21.97 ± 1.74	22.35 ± 1.70	22.73 ± 1.56	< 0.001
Current smoking	698 (42.20%)	471 (28.49%)	361 (21.83%)	312 (18.86%)	< 0.001
Current drinking	661 (39.99%)	351 (21.23%)	306 (18.50%)	229 (13.85%)	< 0.001
Education					0.948
Less than high school	1059 (91.29%)	1107 (91.49%)	1165 (91.95%)	1258 (91.56%)	
At least high school	101 (8.71%)	103 (8.51%)	102 (8.05%)	116 (8.44%)	
Physical activity^a^					< 0.001
Mild	581 (50.09%)	669 (55.29%)	726 (57.30%)	776 (56.48%)	
Moderate	279 (24.05%)	295 (24.38%)	276 (21.78%)	336 (24.45%)	
Vigorous	300 (25.86%)	246 (20.33%)	265 (20.92%)	262 (19.07%)	
SBP, mmHg	148.25 ± 18.34	149.21 ± 17.92	148.67 ± 18.07	148.86 ± 17.39	0.482
DBP, mmHg	88.42 ± 10.79	88.42 ± 10.44	88.12 ± 10.35	88.75 ± 10.49	0.396
Pulse rate, bpm	73.70 ± 14.97	75.31 ± 14.20	76.16 ± 13.76	78.06 ± 14.35	< 0.001
Hcy, μmol/L	18.91 ± 11.39	19.02 ± 12.44	17.79 ± 10.79	16.76 ± 8.67	< 0.001
FPG, mmol/L	5.85 ± 1.23	5.94 ± 1.27	6.06 ± 1.38	6.43 ± 1.85	< 0.001
TC, mmol/L	4.99 ± 1.03	5.10 ± 1.06	5.24 ± 1.12	5.19 ± 1.17	< 0.001
TG, mmol/L	0.84 ± 0.22	1.20 ± 0.28	1.63 ± 0.39	2.91 ± 1.53	< 0.001
HDL-C, mmol/L	1.97 ± 0.46	1.70 ± 0.38	1.54 ± 0.33	1.30 ± 0.30	< 0.001
LDL-C, mmol/L	2.64 ± 0.73	2.91 ± 0.76	3.12 ± 0.81	3.13 ± 0.80	< 0.001
eGFR, mL/min/1.73 m^2^	88.50 ± 19.71	88.04 ± 19.63	88.25 ± 19.98	88.35 ± 20.72	0.927
Serum aspartate aminotransferase, U/L	27.36 ± 10.54	25.71 ± 10.84	25.47 ± 13.76	25.65 ± 10.16	< 0.001
Serum alanine aminotransferase, U/L	17.53 ± 9.94	17.79 ± 11.84	18.40 ± 14.40	20.08 ± 13.07	< 0.001
Serum γ-glutamyltransferase, U/L	30.62 ± 47.38	26.32 ± 34.81	29.02 ± 38.05	35.10 ± 50.97	< 0.001
Diabetes mellitus^b^	167 (10.10%)	200 (12.10%)	259 (15.66%)	417 (25.21%)	< 0.001
CHD	74 (4.47%)	88 (5.32%)	95 (5.74%)	80 (4.84%)	0.367
Stroke	93 (5.62%)	108 (6.53%)	127 (7.68%)	111 (6.71%)	0.128
PAD	47 (2.84%)	54 (3.27%)	66 (3.99%)	45 (2.72%)	0.153
Antihypertensive drugs	1018 (61.55%)	1064 (64.37%)	1113 (67.29%)	1092 (66.02%)	0.004
Lipoprotein-lowering drugs	32 (1.93%)	44 (2.66%)	60 (3.63%)	65 (3.93%)	0.003
Glucose-lowering drugs	43 (2.60%)	58 (3.51%)	82 (4.96%)	111 (6.71%)	< 0.001

*BMI* body mass index; *SBP* systolic blood pressure; *DBP* diastolic blood pressure; *Hcy* homocysteine; *FPG* fasting plasma glucose; *TC* total cholesterol; *TG* triglycerides; *HDL* high-density lipoprotein cholesterol; *LDL-C* low-density lipoprotein cholesterol; *eGFR* estimated glomerular filtration rate; *CHD* coronary heart disease

^a^Physical activity was defined as mild, moderate, or vigorous according to the participant’s personal evaluation

^b^Diabetes mellitus was defined as self-reported physician diagnosis of diabetes or FBG concentration ≥ 7.0 mmol/L or use of glucose-lowering drugs

### Association of VAI with PAD

**Fig. 2:**
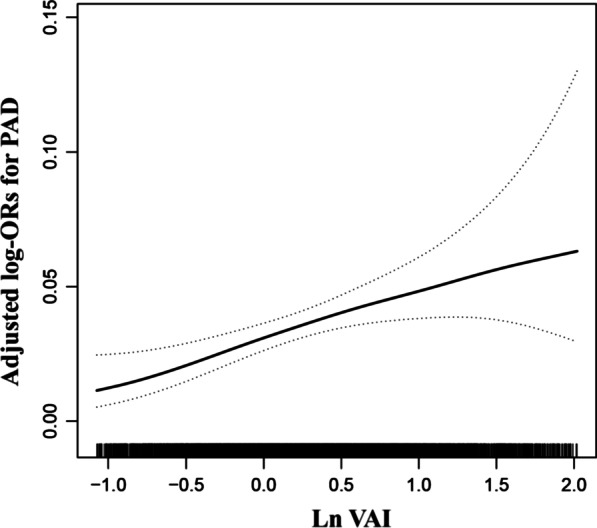
The association between VAI and the prevalence of PAD. The solid line and dashed line represent the estimated values and their corresponding 95% confidence interval, respectively. The adjustment factors included age, sex, BMI, SBP, DBP, diabetes mellitus, stroke, CHD, antihypertensive drugs, lipoprotein-lowering drugs, glucose-lowering drugs, current smoking, current drinking, education, physical activity, Hcy, FBG, TC, LDL, eGFR, serum aspartate aminotransferase, serum alanine aminotransferase, serum γ-glutamyltransferase

**Fig. 3:**
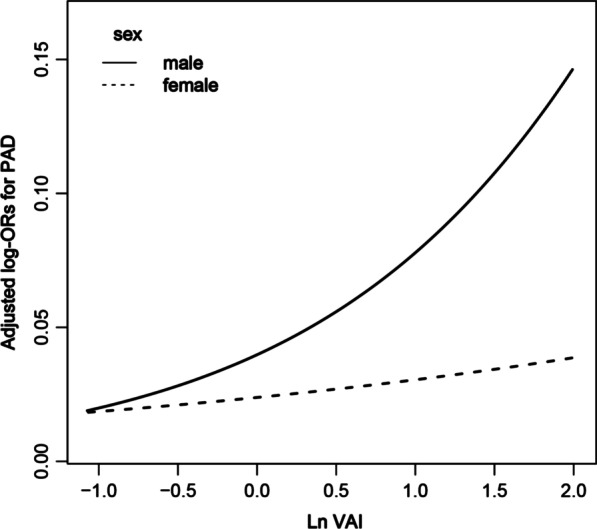
The association between VAI and the risk of PAD by sex. The solid line and dashed line represent the estimated values in male and female, respectively. The adjustment factors included age, BMI, SBP, DBP, diabetes mellitus, stroke, CHD, antihypertensive drugs, lipoprotein-lowering drugs, glucose-lowering drugs, current smoking, current drinking, education, physical activity, Hcy, FBG, TC, LDL, eGFR, serum aspartate aminotransferase, serum alanine aminotransferase, serum γ-glutamyltransferase

**Fig. 4:**
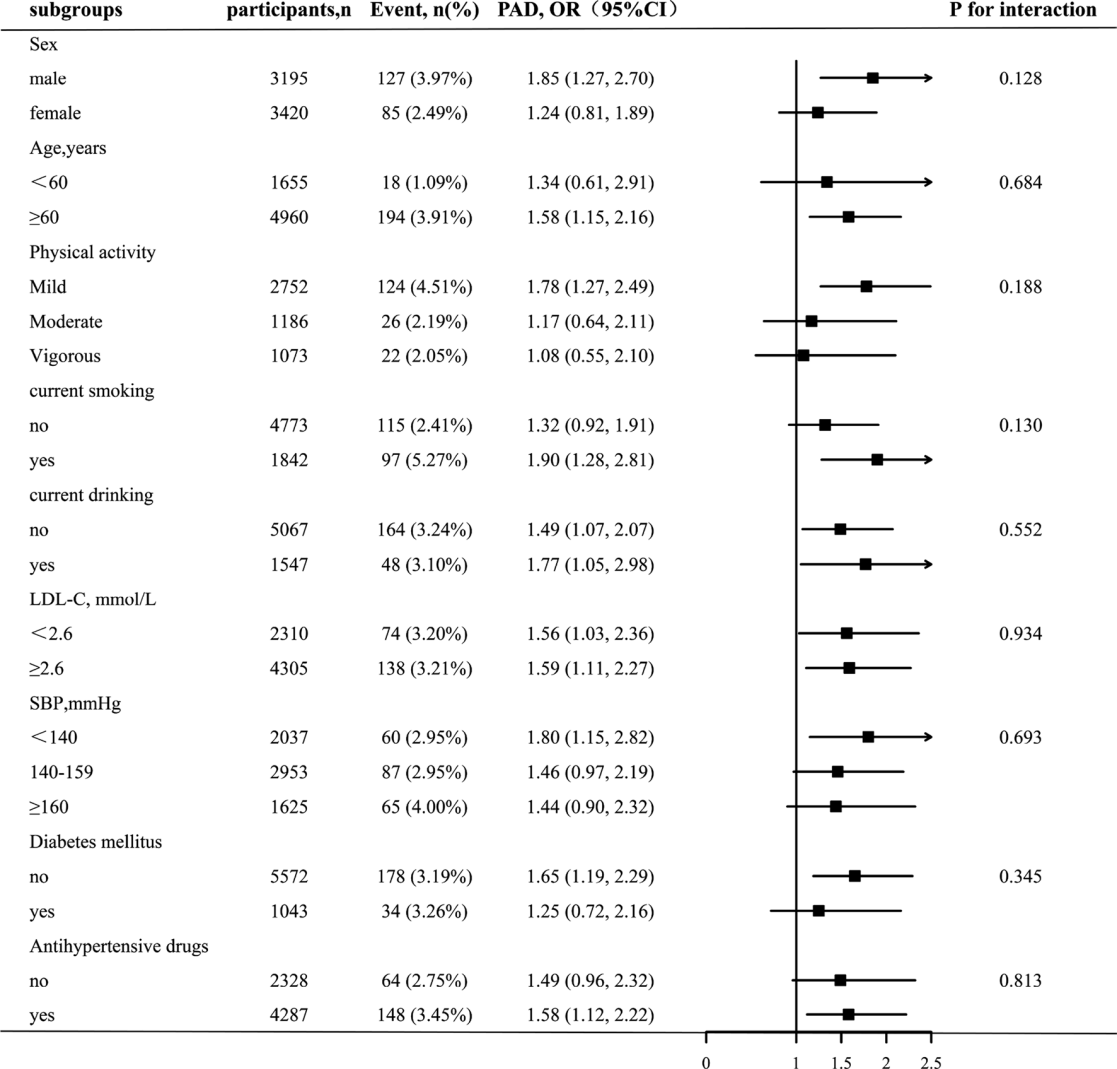
Stratified analyses by potential modifiers of the association between VAI and the prevalence of PAD^**^Each subgroup analysis adjusted for age, sex, BMI, SBP, DBP, pulse rate, diabetes mellitus, stroke, CHD, antihypertensive drugs, lipoprotein-lowering drugs, glucose-lowering drugs, current smoking, current drinking, education, physical activity, Hcy, FBG, TC, LDL, eGFR, serum aspartate aminotransferase, serum alanine aminotransferase, serum γ-glutamyltransferase except for the stratifying variable

Overall, there was a significant positive association between VAI and PAD prevalence (Fig. 2). For per unit increment in LnVAI, the adjusted odds ratios (ORs) of PAD for participants in Models 1–4 were 1.52 (95% confidence interval [CI] 1.23–1.87), 1.71 (95% CI 1.38–2.13), 1.63 (95% CI 1.25–2.11), and 1.55 (95% CI 1.15–2.10), respectively. Accordingly, in the Model 4, when VAI was assessed in quartiles and compared with quartile 1 (< 0.84), the PAD prevalence was higher than that of quartiles 2 (0.84 to < 1.36: OR 1.49; 95% CI 0.92–2.44), 3 (1.36 to < 2.25: OR 1.95; 95% CI 1.14–3.32), and 4 (≥ 2.25: OR 1.93; 95% CI 1.04–3.57) (Table 2). In Model 4, a significantly positive association between VAI and PAD prevalence was found among male patients (OR 2.12, 95% CI 1.46–3.07). Although there were similar trends among female patients, the difference was not statistically significant (OR 1.28; 95% CI 0.85–1.95) (Table 3). Figure 3 shows that the generalized additive model and fitted smoothing curve (penalized spline method) are consistent with multivariate regression models for the different sexes. There was no interaction between sex and VAI (*P* for interaction = 0.128) (Fig. 4).

**Table 2:** Relative odds of PAD according to VAI in different models among normal-weight hypertensive patients

VAI					
PAD	Quartile 1 (< 0.84)	Quartile 2 (0.84 to < 1.36)	Quartile 3 (1.36 to < 2.25)	Quartile 4 (≥ 2.25)	*P* for trend
Participants, *n*	1654	1653	1654	1654	
Cases, *n*	47 (2.84%)	54 (3.27%)	66 (3.99%)	45 (2.72%)	
Model 1	Reference	1.39 (0.92, 2.09)	2.13 (1.42, 3.20)	1.83 (1.16, 2.89)	0.001
Model 2	Reference	1.56 (1.03, 2.37)	2.50 (1.64, 3.80)	2.29 (1.42, 3.68)	< 0.001
Model 3	Reference	1.64 (1.02, 2.64)	2.27 (1.39, 3.72)	2.22 (1.27, 3.90)	0.002
Model 4	Reference	1.49 (0.92, 2.44)	1.95 (1.14, 3.32)	1.93 (1.04, 3.57)	0.030

Values are ORs (95% CIs) unless otherwise indicated. PAD, peripheral arterial disease; VAI, visceral adiposity index

Model 1 was adjusted for age, sex

Model 2 was adjusted for age, sex, BMI, SBP, DBP

Model 3 was adjusted for age, sex, BMI, SBP, DBP, diabetes mellitus, stroke, CHD, antihypertensive drugs, lipoprotein-lowering drugs, glucose-lowering drugs, current smoking, current drinking, education, physical activity

Model 4 was adjusted for age, sex, BMI, SBP, DBP, diabetes mellitus, stroke, CHD, antihypertensive drugs, lipoprotein-lowering drugs, glucose-lowering drugs, current smoking, current drinking, education, physical activity, Hcy, FBG, TC, LDL, eGFR, serum aspartate aminotransferase, serum alanine aminotransferase, serum γ-glutamyltransferase

**Table 3:** Relative odds of PAD according to VAI in different models among normal-weight hypertensive patients in different sex

VAI Index	Participants, *n*	Cases, *n*	PAD, OR (95%CI)			
			Model 1	Model 2	Model 3	Model4
Male						
Per 1 unit increase	3195	127 (3.97%)	1.65 (1.26, 2.17)	1.94 (1.46, 2.59)	1.96 (1.38, 2.79)	2.12 (1.46, 3.07)
Quartiles						
Q1 (< 0.64)	799	18 (2.25%)	Reference	Reference	Reference	Reference
Q2 (1.17 to < 1.76)	798	37 (4.64%)	1.98 (1.11, 3.54)	2.20 (1.22, 3.94)	1.89 (0.95, 3.76)	1.94 (0.97, 3.90)
Q3 (1.76 to < 2.74)	799	37 (4.63%)	2.18 (1.22, 3.89)	2.56 (1.41, 4.62)	2.32 (1.16, 4.63)	2.27 (1.13, 4.60)
Q4 (≥ 2.74)	799	35 (4.38%)	2.83 (1.57, 5.09)	3.67 (1.99, 6.77)	3.11 (1.49, 6.49)	3.26 (1.53, 6.93)
Female						
Per 1 unit increase	3420	85 (2.49%)	1.34 (0.96, 1.86)	1.44 (1.03, 2.03)	1.24 (0.83, 1.86)	1.28 (0.85, 1.95)
Quartiles						
Q1 (< 1.17)	855	14 (1.64%)	Reference	Reference	Reference	Reference
Q2 (1.17 to < 1.76)	855	23 (2.69%)	1.62 (0.82, 3.20)	1.70 (0.86, 3.36)	1.20 (0.54, 2.68)	1.23 (0.54, 2.75)
Q3 (1.76 to < 2.74)	855	23 (2.69%)	1.80 (0.91, 3.54)	1.85 (0.93, 3.66)	1.86 (0.86, 4.02)	1.89 (0.86, 4.13)
Q4 (≥ 2.74)	855	25 (2.92%)	2.14 (1.09, 4.17)	2.39 (1.21, 4.71)	1.77 (0.80, 3.92)	1.82 (0.81, 4.07)

Model 1 was adjusted for age

Model 2 was adjusted for age, BMI, SBP, DBP

Model 3 was adjusted for age, BMI, SBP, DBP, diabetes mellitus, stroke, CHD, antihypertensive drugs, lipoprotein-lowering drugs, glucose-lowering drugs, current smoking, current drinking, education, physical activity

Model 4 was adjusted for age, BMI, SBP, DBP, diabetes mellitus, stroke, CHD, antihypertensive drugs, lipoprotein-lowering drugs, glucose-lowering drugs, current smoking, current drinking, education, physical activity, Hcy, FBG, TC, LDL, eGFR, serum aspartate aminotransferase, serum alanine aminotransferase, serum γ-glutamyltransferase

### Stratified analyses by additional factors

Stratified analyses were performed to explore the potential interactions between VAI and PAD prevalence, as shown in Fig. 4. None of the variables, including sex (males vs. females), age (< 60 vs. ≥ 60), physical activity (mild, moderate, or vigorous), current smoking status (no vs. yes), drinking status (no vs. yes), diabetes mellitus (no vs. yes), SBP (< 140, 140–159, or ≥ 160 mm Hg), LDL-C (< 2.6 vs. ≥ 2.6 mmol/L), and antihypertensive drugs (no vs. yes) significantly modified the association between VAI and PAD prevalence (*P* values for all interactions > 0.05).

## Discussion

In the present study, we performed a large cross-sectional study using data from the China H-type Hypertension Registry. The results demonstrated that VAI was positively associated with PAD in normal-weight patients with hypertension. In addition, a positive association between higher VAI levels and PAD prevalence was found among men than women.

Most previous studies assessing the effects of VAI on cardiometabolic risks [26, 27] established a statistical correlation between higher VAI levels and a higher prevalence of arterial stiffness [28], coronary heart disease [29], hypertension [30], and cardiovascular mortality [31, 32]. However, there have been few studies on VAI and PAD. Only Wung et al. explored the relationship between obesity-related indicators and PAD in 1872 patients with type 2 diabetes. The results showed that an increase in VAI levels was related to PAD prevalence [17]. However, no sex difference was found in Wung et al.’s research due to differences in the research population, design, and sample size.

The mechanism between VAI and PAD in normal-weight patients with hypertension may be explained by insulin resistance (IR) and inflammation. Previous studies have shown that even people with normal weight may be metabolically obese [13]. A more sensitive VAI can then replace the metabolic obesity produced at this time, and we know that VAI shows the strongest correlation between IR and lipid metabolism [33]. Visceral adipocytes stimulate the secretion of inflammatory factors, such as interleukin-6 (IL-6), tumor necrosis factor-alpha (TNF-α), and fat factors [34, 35]; thus, causing the production of reactive oxygen species in arterioles. Nitric oxide production and consumption decrease and increase under the combined action of inflammatory factors and reactive oxygen species, respectively [36]. Therefore, pro-inflammatory cytokines and reactive oxygen species from obesity can produce peripheral IR and directly affect the endothelium, leading to endothelial dysfunction, and atherosclerosis cascade reaction [37]. The prevalence of diabetes and early abnormal glucose metabolism in men is higher than that in women because insulin sensitivity differs between men and women [38, 39]. Therefore, men are more likely to have an increased risk of IR than women. Therefore, the positive correlation between VAI and PAD was more obvious in men.

The present study provides an opportunity to explore the dose–response relationship between VAI and PAD in normal-weight patients with hypertension. The data used were obtained from a large-scale observational study of the China H-type Hypertension Registry Study. Our results provide new insights into this field. To our knowledge, this is the first study to explore the relationship between VAI and PAD in normal-weight patients with hypertension and to find a positive correlation between them. The results showed that an increase in VAI levels was related to PAD prevalence. Second, according to related research, even in the general population, obesity is strongly correlated with cardiovascular disease and death [40]. BMI is usually used to evaluate obesity [41]. Nevertheless, BMI is limited in that it cannot distinguish between muscle and fat content and cannot provide body fat distribution [42, 43]. Obesity is a metabolic disease. However, some normal-weight people may have metabolic disorders similar to those with obesity. These individuals are metabolically obese with normal weight. At the same time, related studies show that the population, as mentioned earlier, accounts for 20% of the normal-weight population [13, 14]. When compared with BMI, visceral fat can better reflect metabolic changes [15]. The VAI, a simple index calculated using blood lipid, WC, and BMI, can be used as a simple biomarker of body fat distribution and metabolic disorders and is closely related to visceral fat measured using MRI [16]. Finally, our results showed significant sex differences, with a positive association between higher VAI levels and PAD prevalence among males than females. The fat distribution differs between men and women owing to differences in sex hormone levels [44]. The decrease in estrogen levels in postmenopausal women leads to the accumulation of adipose tissue in the center/viscera [45–47], where VAI stands for visceral fat. All the women in this study were postmenopausal; therefore, women had higher VAI levels than men. However, there was no significant correlation between VAI and PAD in women. A possible reason for this result may be that, compared with women, men have more risk factors for PAD, such as smoking. Further research is needed to confirm the relationship between VAI and PAD prevalence, and our study results are only in the generated hypothesis stage.

### Limitations

The limitations of the present study should be noted. First, although we adjusted for most of the covariates as much as possible, there may still be unmeasured and residual confounding factors. Second, all the participants in this study were patients with hypertension in Southern China; thus, our conclusions may not be generalizable to different populations. In addition, because this was a limitation of a cross-sectional study, we could not determine the causality and long-term clinical results between them.

## Future directions

In clinical practice, clinicians can closely monitor the VAI level of normal-weight patients with hypertension and observe PAD-related signs in patients with high VAI. At the same time, this study also emphasizes the important role of visceral obesity in the occurrence and development of PAD. Therefore, further large-scale prospective cohort studies are needed to explore the occurrence and development of VAI and PAD in normal-weight patients with hypertension and encourage researchers to dissect the molecular mechanisms involved.

## Conclusions

In conclusion, our cross-sectional study demonstrated a positive association between higher VAI levels and PAD prevalence in normal-weight participants among males than females. Therefore, in clinical practice, more attention should be paid to the VAI levels of normal-weight men with hypertension.

## Data Availability

The datasets used and/or analyzed in the current study are available from the corresponding author upon reasonable request.
